# Varying Effects of Straw-Returning Methods on Soil Microbial Diversity and Community Composition in Northeast China

**DOI:** 10.3390/microorganisms13081749

**Published:** 2025-07-26

**Authors:** Yitao Zhang, Yuxian Wang, Zhanbin Sun

**Affiliations:** 1 Institute of Geographic Sciences and Natural Resources Research, Chinese Academy of Sciences, Beijing 100101, China; zhangyt@igsnrr.ac.cn; 2 Qiqihar Branch of Heilongjiang Academy of Agricultural Sciences, Qiqihar 161006, China; 3 School of Light Industry Science and Engineering, Beijing Technology and Business University, Beijing 100048, China

**Keywords:** straw-returning, soil microbial diversity, soil microbial community composition, *Trichoderma*, *Chaetomium*, *Streptomyces*

## Abstract

Straw-returning is an effective way to improve straw utilization efficiency and reduce environmental pollution. Various straw-returning methods exist; however, their effects on soil microbial diversity and community composition in cool regions have been little studied. This study investigated the changes of soil microbial diversity and community composition under three straw-returning methods, i.e., straw mulching, straw mulching and overturning, straw crushed and mixed, as compared to straw removal as control. The results showed that straw-returning could alter the soil microbial community composition and abundance compared with straw removal. Alpha diversity analysis showed that straw mulching treatment, and straw crushed and mixed treatment significantly increased the diversity of both soil bacteria and fungi compared with straw mulching and overturning treatment. Moreover, this study preliminarily screened *Trichoderma*, *Chaetomium* and *Streptomyces* as potential straw-degrading microorganisms. This study provides basis for further enhancement of straw degradation by using soil microorganisms and sheds light on future work for improving straw degradation efficiency.

## 1. Introduction

Crop straws, the stems and leaves left after harvest, were long regarded as agricultural waste but are now recognized as valuable sources of organic matter and minerals [1]. Traditionally, large quantities of crop straw were burned in open fields or used as household fuel, leading to significant air pollution—for instance, 100 million tons annually in China and 80 million tons in India. Due to the large-scale, highly intensive crop production, China produces 700–900 million tons straw per year (National Bureau of Statistics data, http://www.stats.gov.cn/), which accounts for approximately 30% of the global total straw quantity [2]. The burning of straw was a top environmental problem for many years [3,4] and thus has been banned in national/provincial environmental policies and action plans [5]. While straw retention in fields is now mandated, the optimal management method for sustainable agriculture and ecological health remains unclear [6,7].

There are several ways of straw handling in the field, also widely called straw-returning to the soil. These include straw mulching, straw mulching and overturning, straw crushed and mixed, straw manure, and straw biochar, etc. [8,9]. As compared to removing the straw from the field, overall, long-term straw-returning was found to enhance soil fertility and crop yield, improve soil micro-ecology, and avoid environmental pollution caused by incineration [6,7]. However, different straw-returning methods potentially have differing impacts on soil physicochemical properties, crop yield and soil microbial community diversity and structure. In a recent study examining the effects of different straw-returning methods on corn growth and yield, Xu [9] found that they had differing impacts on corn height, stem diameter and leaf area. Straw mulching significantly increased corn yield as compared to straw mulching and overturning, and straw crushed and mixed. Huang et al. [5] found that cornstalk returning after carbonization had a stronger effect on soil physicochemical properties and corn growth than cornstalk returning without carbonization. In another study, Li et al. [10] found that although all straw treatments significantly increased corn yield as compared to the null straw treatment, straw mulching resulted in dramatically larger amounts of cumulative soil CO_2_ emissions than the treatments with straw-returning at either shallow or deep soil depths. These studies provide good knowledge for practical soil straw management but also points to the need of in-depth understanding of the mechanisms behind the observed effects, particularly relevant biological mechanisms. 

Soil microbes play important roles in straw degradation and resource utilization [11]. The main mechanisms for soil microbe degradation are related to the degradation of cellulose polymers, which are the main component of the straw [12]. Many microbes, including fungi such as *Trichoderma*, *Aspergillus* and *Chaetomium*, and bacteria such as *Bacillus* and *Streptomyces*, have been reported to be involved in straw degradation [11,13,14,15]. These findings show the promise to enhance straw degradation by utilizing microbes and providing them with optimal environmental conditions. Liu et al. [16] found that different rates of straw-returning dramatically influenced bacterial community in a lime concretion black soil. Likewise, when different methods are used for straw-returning, they can also hypothetically affect soil microbial diversity and community composition due to their varying effects on the environmental conditions for the microbes [17]. Such microbiological effects should be assessed, because the understanding of different straw-returning methods effects on soil microbial diversity and community compositions can provide important science basis for further improving the resource utilization rate of the straw.

In Northeast China, the exploration of best straw-returning method is challenging but especially needed. This region constitutes about a quarter of the national land-based food production in China, but the long and cool non-growing season results in slower degradation of crop straws as compared to many other high-production regions. This demands appropriate farming practices for maximizing the resource utilization rate of straws. Here we studied three different straw-returning methods, i.e., straw mulching, straw mulching and overturning, and straw crushed and mixed, and compared them to a scenario with no straw-returning. The above three straw retuning methods were commonly used in regional farming systems and had strong practicality. The objective of this study was to examine the effects of the straw-returning methods on soil microbial diversity and community composition, as a basis for improving the straw degradation ability by soil microbes.

## 2. Materials and Methods

### 2.1. Field Site

The field experiment was conducted in the Fulaerji District, Qiqihaer City, Heilongjiang Province, China (123°41′ E, 47°16′ N). The region is part of the Northeast China Plain, the largest plain in China. The region has a temperate monsoon climate, with short and rainy summers, and long and cool winters. The experimental site had an annual precipitation of 400–500 mm that mainly occurred as rainfall during July to September. Annual mean air temperature was 3.2 °C, with the average temperature of −18.7 °C in January and 22.8 °C in July. The experimental soil was carbonate chernozem, with the following properties measured at the start of the experiment: organic matter, 24.30 g kg^−1^; total nitrogen, 1.28 g kg^−1^; total phosphorus, 0.91 g kg^−1^; total potassium, 5.00 g kg^−1^; alkali-hydrolysable nitrogen, 100.1 mg kg^−1^; plant-available phosphorus, 49.39 mg kg^−1^; and rapidly available potassium, 271.5 mg kg^−1^. The experimental crop was corn (Nen Dan 19), with the plant density of 67,500 plants per ha. 

### 2.2. Experimental Design and Soil Sampling

Four straw-returning treatments were set up in a large, 13.2 ha experimental field since 2016, and soil samples were collected after the corn harvest in autumn 2022. The treatments were: (1) straw removal (SR); (2) straw mulching (SM); (3) straw mulching and overturning (SO); and (4) straw crushed and mixed (SC). Straws were chopped (<10 cm) by using straw pulverator. In the SR treatment, all straw was removed post-harvest. For SM, chopped straws were applied to the soil surface. In SO, chopped straws were mulched and then incorporated to a depth nearly 30 cm before soil freezing. In SC, chopped straws were mixed into the 0–30 cm soil layer using machinery. Each treatment covered an area of 3.3 ha. The experimental field was flat and known to have homogenous soil properties prior to the field treatments. For each treatment, three replicated soil samples were collected from different sections of the field, at the depth of 0–20 cm, for further molecular and bioinformatic analyses. Each soil sample comprised multiple soil cores collected from across the field section.

Using block comparison for each treatment, and each treatment plot was 0.33 ha. A compound fertilizer (14% N, 22% P_2_O_5_, 14% K_2_O) was applied once in spring at a rate of 750 kg ha^−1^. Mechanical precision sowing was implemented followed by 30 mm sprinkler irrigation after planting. Chemical weed control was conducted at the four-leaf stage of corn. When sampling each treatment block, three soil samples were collected respectively, with each soil sample being a composite mixture from five sampling points. All the blocks are randomized to analysis the micro-ecological effects. 

### 2.3. Soil DNA Extraction and PCR Amplification

Soil DNAs were extracted using the FastDNA^TM^ Spin Kits (MP, Santa Ana, CA, USA), following the manufacturer’s instructions. The quality and integrity of the extracted DNAs were measured using NanoDrop^TM^ 2000 (Thermo Scientific, Wilmington, NC, USA) and agarose gel electrophoresis, respectively. 

Primer pairs of 338F (5′-ACTCCTACGGGAGGCAGCAG-3′) and 806R (5′-GGACTACHVGGGTWTCTAAT-3′) were used to amplify the V3-V4 region of 16S rRNA encoding gene among the extracted DNA [18]. A 20 μL system for amplification of V3-V4 region was prepared from 10 ng template DNA, 0.4 μL FastPfu polymerase, 0.8 μL forward primers and reverse primers, 2 μL dNTPs, 4 μL buffer, 0.2 μL BSA, and residual ddH_2_O. Primer pairs of ITS1F (5′-CTTGGTCATTTAGAGGAAGTAA-3′) and ITS2R (5′-GCTGCGTTCTTCATCGATGC-3′) were used to amplify ITS regions of the extracted DNA [19]. The 20 μL PCR reaction system for amplification of ITS region was comprised of 10 ng template DNA, 0.2 μL rTaq polymerase, 0.8 μL forward primers and reverse primers, 2 μL dNTPs, 2 μL buffer, 0.2 μL BSA, and residual ddH_2_O. The PCR reaction for amplifying both the V3-V4 and ITS regions were conducted on ABI GeneAmp^®^ 9700 (Waltham, MA, USA). Agarose gel electrophoresis was used to detect PCR products, and AxyPrep DNA Gel Extraction Kit (Axygen, Union City, CA, USA) was used to recover the products. Set amplification of sterilized ddH_2_O as negative control.

### 2.4. Illumina MiSeq Sequencing

TruSeq DNA Sample Prep Kit (Illumina, San Diego, CA, USA) was used to construct MiSeq sequencing libraries. A paired-end (300) sequencing strategy was used for conducting MiSeq sequencing on an Illumina MiSeq platform (Illumina) by the Majorbio Co. Ltd. (Shanghai, China). The FLASH software 1.2.11 and Fastp software 0.20.0 were used to assemble, filtering and quality control of raw sequence reads produced by the MiSeq sequencing [20,21,22]. Low quality raw sequencing reads, which contained nitrogen base, and the sequence length and average quality score lower than 50 bp and 20, respectively, were discarded. Chloroplast and mitochondrial sequences were also removed. The number of sequences among treatments were normalized and then conducted for further bioinformatic analysis. 

### 2.5. Bioinformatic Analysis

The UPARSE software (version 7.1) was used to cluster operational taxonomic units (OTU) with 97% similarities [23]. Moreover, the taxonomy of OTU was analyzed by using the RDP classifier (version 2.13) [24]. The classification database for bacteria and fungi is silva138/16s_bacteria and unite8.0/its_fungi, respectively. 

Analysis of the alpha diversity of soil bacteria and fungi community among different treatments, which reflects their diversity and richness, was conducted by evaluating the following indices: sobs, Shannon, Simpson, ace, and chao. Any significant differences in alpha diversity among different treatments were detected through a Wilcoxon rank-sum test. A Principal Coordinates Analysis (PCoA) with the R package (version 3.3.1) was used for beta diversity analysis to analyze the similarities or differences of soil bacteria and fungi communities among the different treatments by using bray-curtis dissimilarity. The significance of differences in soil bacteria and fungi abundance was analyzed by using a one-way ANOVA analysis, as well as a Linear discriminant analysis Effect Size (LEfSe) analysis, with standard of Linear Discriminant Analysis (LDA) score higher than 2 [25]. The used confidence intervals is 0.95.

## 3. Results

### 3.1. Illumina MiSeq Sequencing and OTU Cluster Analysis

For all the soil samples, the Illumina MiSeq sequencing generated 1,135,090 sequences for bacteria and 997,897 sequences for fungi. The length of sequencing averaged 417 bp for bacteria and 237 bp for fungi. With regard to the different straw treatments, 3319, 3193, 2890 and 3117 bacterial OTUs were clustered in SR, SM, SO and SC, respectively. For fungi, 725, 766, 558 and 800 OTUs were clustered in the four treatments, respectively.

### 3.2. Analysis of Soil Bacterial and Fungal Community Composition

For soil bacteria community composition at the phylum level, the four straw treatments had the same top four phyla, i.e., Actinobacteriota, Proteobacteria, Chloroflexi and Acidobacteriota. The proportion of Actinobacteriota was the highest phyla in all the four treatments. The proportion of Chloroflexi was higher than Acidobacteriota in SM and SO treatment, while in SR and SC treatment, the proportion of Acidobacteriota was higher than Chloroflexi. The relative proportion of Firmicutes in SO treatment was higher than in other three treatments (Figure S1).

**Figure 1:**
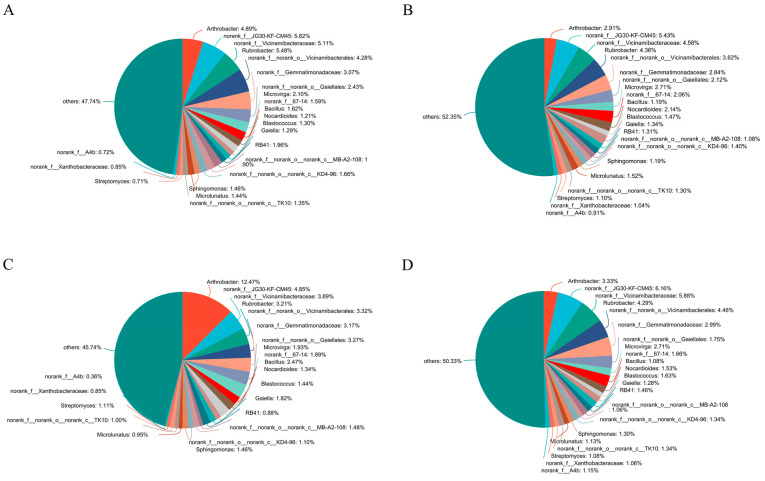
Soil bacterial community composition at the genus level. (**A**) SR, (**B**) SM, (**C**) SO, (**D**) SC. SR: straw removal; SM: straw mulching; SO: straw mulching and overturning; and SC: straw crushed and mixed.

For soil bacteria community composition at the genus level, SO treatment exhibited quit different genus compared with other three treatments (Figure 1). The top genus in the SO treatment was *Arthrobacter*, while in other three treatments, the top genus was norank_f__JG30-KF-CM45. Besides the top genus, the relative proportion of some genera were different among the four treatments. The relative proportion of *Streptomyces* in three straw-returning treatments were higher than SR control treatment. In contrary, the relative proportion of *Rubrobacter* was higher in SR control treatment than other three straw-returning treatments. The relative proportion of *Microvirga* was higher in SM and SC treatment than in SR and SO treatment. In addition, the relative proportion of *Bacillus* and *Gaiella* in SO treatment was higher than in other three treatments. 

For soil fungal community composition at the phylum level, the top three phyla in all the four treatments were the same, i.e., Ascomycota, Basidiomycota and Mortierellomycota. The relative proportion of Chytridiomycota in SR and SC treatment was higher than in SM and SO treatments (Figure S2).

**Figure 2:**
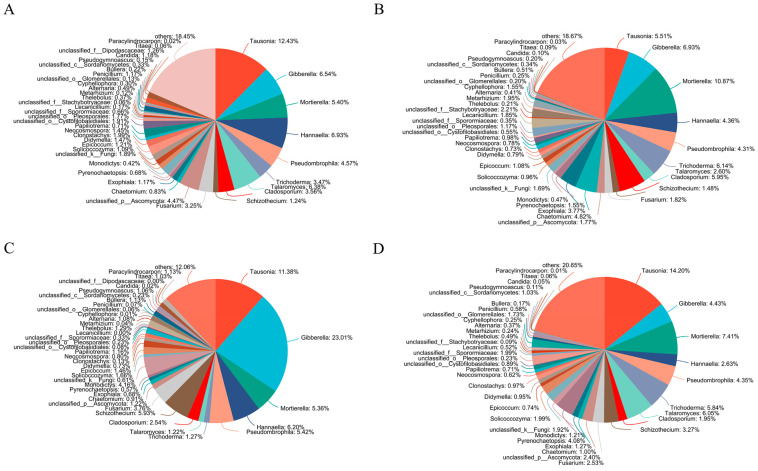
Soil fungal community composition at the genus level. (**A**) SR, (**B**) SM, (**C**) SO, (**D**) SC. SR: straw removal; SM: straw mulching; SO: straw mulching and overturning; and SC: straw crushed and mixed.

Similar to bacteria, the straw treatments also had different soil fungal community compositions at the genus level (Figure 2). In SR and SC treatments, the top genus was *Tausonia*. While in SM and SO treatments, the top genus was *Mortierella* and *Gibberella*, respectively. The relative proportion of *Trichoderma* was higher in SM and SC treatment than in SR and SO treatment. In SR treatment, the relative proportion of *Didymella* was higher than other treatments. In SM treatment, the relative proportion of *Cladosporium*, *Chaetomium*, *Lecanicillium*, *Metarhizium* and *Cyphellophora* was higher than other treatments. In SO treatment, the relative proportion of *Schizothecium*, *Monodictys*, *Thelebolus* and *Alternaria* was higher than other treatments. In SC treatment, the relative proportion of *Pyrenochaetopsis* was higher than other treatments. 

### 3.3. The Difference of Soil Bacterial and Fungal Community Analysis

**Figure 3:**
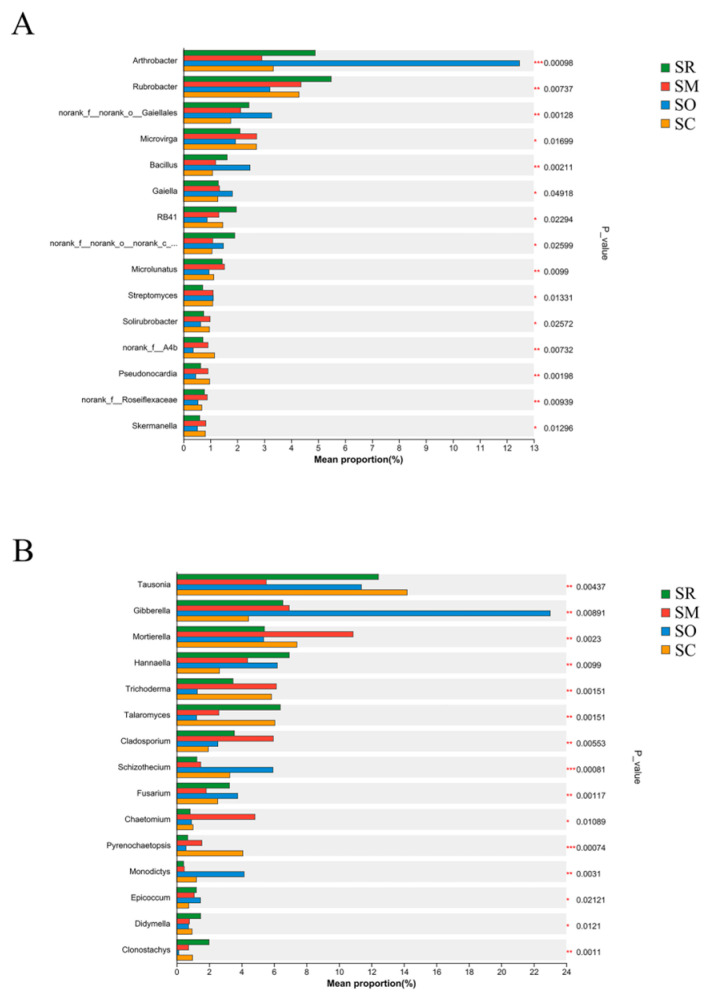
Differences in the community abundance at the genus level for: (**A**) soil bacterial community and (**B**) soil fungal community. *X*-axis shows the proportion of community abundance, and *y*-axis shows bacterial genera. SR: straw removal; SM: straw mulching; SO: straw mulching and overturning; and SC: straw crushed and mixed. * indicates a significant level at *p* < 0.05, ** at *p* < 0.01, and *** at *p* < 0.001.

The Kruskal-Wallis H test revealed significant differences in the top 15 abundant soil bacteria and fungi genera among the four straw treatments (Figure 3). For soil bacteria, the abundance of genera of *Arthrobacter*, norank_f__norank_o__Gaiellales, *Bacillus*, *Microlunatus* and *Pseudonocardia* differed significantly at the level of *p* < 0.01 among the straw treatments (Figure 3A). For soil fungi, the abundance of genera of *Tausonia*, *Gibberella*, *Mortierella*, *Hannaella*, *Trichoderma*, *Talaromyces*, *Cladosporium*, *Schizothecium*, *Fusarium* and *Monodictys* differed significantly at the level of *p* < 0.01, and the *p* values of genera *Schizothecium*, unclassified_p__Ascomycota and *Pyrenochaetopsis* were even lower than 0.001 (Figure 3B).

**Figure 4:**
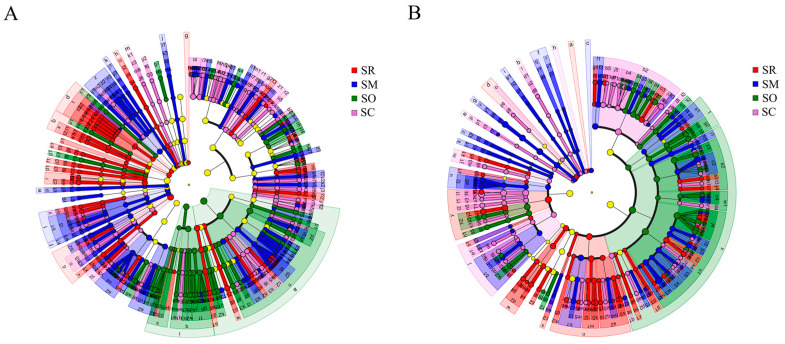
Linear discriminant analysis Effect Size (LEfSe) analysis of the soil bacterial and fungal community composition: (**A**) soil bacterial community and (**B**) soil fungal community. Taxon units from the inside to the outside indicate phylum, class, order, family and genus, respectively. SR: straw removal; SM: straw mulching; SO: straw mulching and overturning; and SC: straw crushed and mixed. Different letters represent different taxon units in phylum, class, order, family and genus.

The LEfSe analysis (LDA score > 2) showed significant differences in soil bacteria and fungi at the levels from phylum to genus among the four straw treatments. In soil bacteria, the numbers at each taxonomy level were different among the treatments (Figure 4A). Specifically, the soil bacteria of SR contained 5 phyla, 10 classes, 18 orders, 29 families and 44 genera. In SM, there were 5 phyla, 6 classes, 18 orders, 37 families and 65 genera. There were 2 phyla, 5 classes, 14 orders, 27 families and 42 genera in SO, and 1 phylum, 3 classes, 19 orders, 30 families and 44 genera in SC. Compared to bacteria, the corresponding numbers were lower for soil fungi. Specifically, the soil fungi of SR contained 2 phylum, 6 classes, 13 orders, 27 families, 45 genera. The treatment SM had 3 phyla, 8 classes, 13 orders, 28 families and 46 genera, SO had 2 classes, 7 orders, 14 families, 32 genera, but without phylum, and SC had 4 phyla, 8 classes, 17 orders, 34 families and 53 genera (Figure 4B).

### 3.4. Alpha Diversity Analysis for Soil Bacterial and Fungal Communities

**Figure 5:**
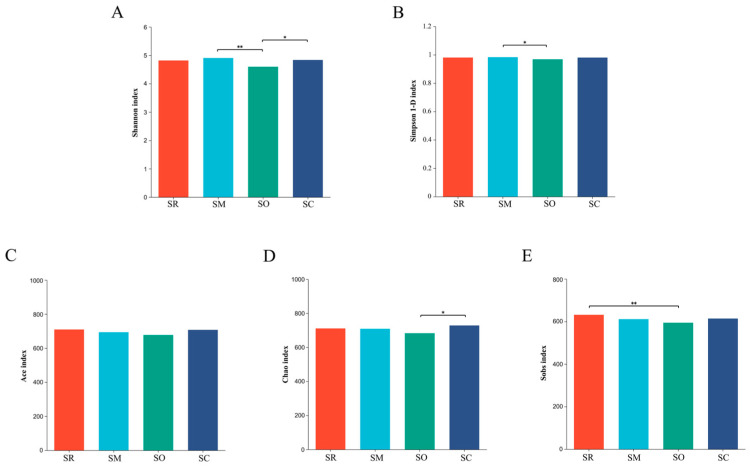
Alpha diversity analysis of soil bacteria at the genus level. *X*-axis shows different treatments, and *y*-axis shows different indices. (**A**) Shannon index, (**B**) Simpson index, (**C**) Ace index, (**D**) Chao index, and (**E**) Sobs index. SR: straw removal; SM: straw mulching; SO: straw mulching and overturning; and SC: straw crushed and mixed. * indicates a significant level at *p* < 0.05, ** at *p* < 0.01.

**Figure 6:**
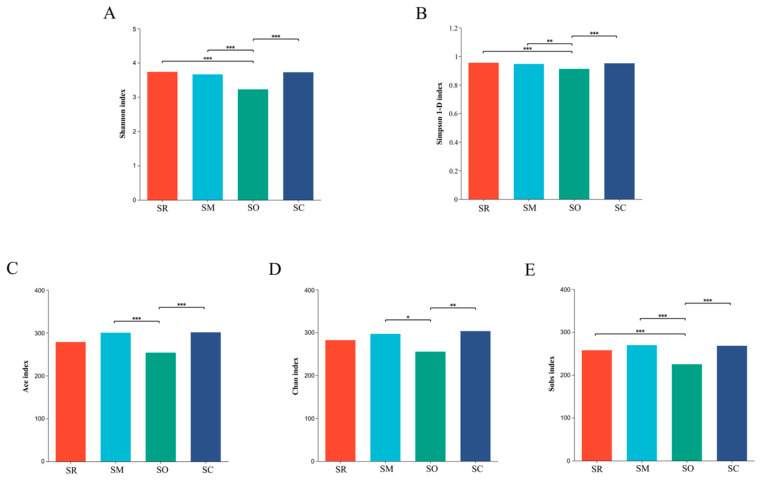
Alpha diversity analysis of soil fungi at the genus level. *X*-axis shows different treatments, and *y*-axis shows different indices. (**A**) Shannon index, (**B**) Simpson index, (**C**) Ace index, (**D**) Chao index, and (**E**) Sobs index. SR: straw removal; SM: straw mulching; SO: straw mulching and overturning; and SC: straw crushed and mixed. * indicates a significant level at *p* < 0.05, ** at *p* < 0.01, and *** at *p* < 0.001.

Indices of Shannon and Simpson 1-D were used to reflect the microbial community diversity. For soil bacterial community, the Shannon index and Simpson 1-D index in the SO treatment was significantly lower than in SM, revealing that the bacterial diversity was altered by different straw-returning methods (Figure 5A,B). Similar results were found in soil fungal communities, SO also had significantly lower Shannon and Simpson 1-D index than the other three treatments (Figure 6A,B).

Indices of sobs, ace and chao represent the richness of microbial communities. For soil bacterial community, all the three indices in the SO treatment were lower than other treatments (Figure 5C–E). For fungal communities, all the three indices in SO were significantly lower than in the other treatments. The results reveal that the straw-returning method influenced the richness of the fungal communities (Figure 6C–E).

### 3.5. Beta Diversity Analysis for Soil Bacterial and Fungal Communities

**Figure 7:**
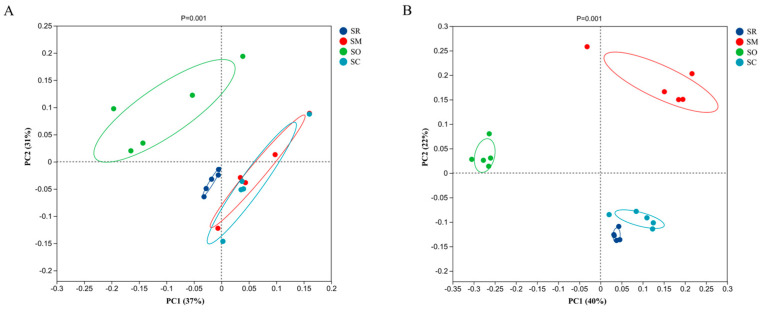
Principal Coordinates Analysis (PCoA) of soil bacterial and fungal communities. *X*-axis and *y*-axis show variance in the community structures of intestinal bacteria. (**A**) Bacterial community, (**B**) Fungal community. SR: straw removal; SM: straw mulching; SO: straw mulching and overturning; and SC: straw crushed and mixed.

The PCoA analysis was conducted to analyze the microbial community composition among different straw treatments. For the analysis on soil bacteria, the PC1 and PC2 values of 37% and 31%, respectively (Figure 7A), reveal the variability of the bacterial communities. The PCoA results showed that SR and SO were separated from SM and SC, which meant that straw-returning could influence the bacterial community composition compared with straw removal. Moreover, straw mulching and overturning could impact the bacterial community composition compared with other straw-returning methods. In contrast, the treatment of straw crushed and mixed and the treatment of straw mulching had similar bacterial community compositions.

For soil fungal community composition, PC1 (40%) and PC2 (22%) represent the variability of soil fungal communities (Figure 7B). The PCoA results showed that all the four straw treatments were separated from each other, which meant that all the three straw-returning methods could influence the soil fungal communities compared with straw removal. Moreover, the three straw-returning methods had different influences on the soil fungal community composition.

## 4. Discussion

Straw-returning is an important way to reduce resource wasting and environmental pollution, meanwhile achieving straw recycling and benefiting sustainable agricultural development [1]. However, the slow process of straw degradation in cold non-growing seasons greatly influences the wild application of straw-returning in northeast China and other high latitude and altitude regions. Therefore, the exploration of good methods for accelerating straw degradation can significantly help promote the application of straw-returning [6,7]. Studies showed that some microorganisms had the ability to degrade the main component of straw, including cellulose [12]. Hence, investigating the impact of straw-returning on soil microbial community to identify optimal straw-returning method for accelerating straw degradation in the soil is helpful for achieving the ultimate goal of straw utilization. In this present study, straw mulching and overturning was lower in soil microbial diversity and richness as compared with straw mulching, straw crushed and mixed, and straw removal. The findings of this study stress straw mulching and overturning as an important technology for straw handling, help reveal the microbial mechanisms of straw handling effects on its degradation, and also provides an important scientific basis for the further selection of useful microorganisms in order for enhanced straw degradation through microbial intervention. 

Alpha diversity analysis found that the three different straw-returning methods exhibit different alpha diversity. Compared with SO treatment, SM and SC treatments significantly increased the alpha diversity both in soil bacteria and fungi. The low alpha diversity in SO treatment might due to the relative anaerobic environment caused by overturning the chopped straws to nearly 30 cm depth soil, and thereby soil microorganisms in SO treatment were difficult to utilize the overturned straws. While in SM and SC treatments, soil microorganisms in either soil surface or mixed into soil had the chance to utilize the chopped straws, therefore remarkably increased the alpha diversity compared with SO treatment.

Besides alpha diversity, the three different straw-returning methods could also influence soil microbial community composition and abundance. Compared with SR and SM treatments, both SO and SC treatments overturned the soil, although SO treatment was overturned the chopped straws below the soil, and SC treatment was mixed the chopped straws into the soil. Both SO and SC treatments might affect the soil oxygen availability, thereby influence the soil microbial community composition and abundance. Previously studied found that oxygen availability could affect the soil microbial community. Miguel et al. found that in the process of decomposition, the oxygen availability could significantly influence the changes of bacterial community composition [26]. Murase et al. found oxygen availability was essential in shaping the community of microeukaryotic [27]. Moreover, different straw-returning methods could form relative soil microclimate, which might also due to the alteration of soil microbial community composition and abundance among different treatments. Castaño et al. found in a Mediterranean pine forest, the soil fungal communities were affected by soil microclimate [28]. Similarly, changes of soil microclimate under plastic mulches influence soil microbial communities [29].

Straw particle size is also other important factor that influence the soil microbial community. Different straw particle size in the soil could provide different accessible surface and available nutrients for microbial community usage [30]. Zhang et al. found that different straw size and amount could regulate the microbial community composition and microbial growth, thereby influence the straw decomposition [31]. In this study, we only choose a commonly practical used size in the field for experiment. In future, we can set several sizes of straw as treatments and investigate the best straw size for enriching the microbial community and improving straw degradation. 

The abundance of several fungi was significantly improved after straw-returning compared with straw removal, and some of the fungi was previously reported to be involved in straw degradation. *Trichoderma* is commonly known as a beneficial fungus in agriculture that can help control plant diseases and promote plant growth [32,33,34]. Earlier studies reported that *Trichoderma* species could produce straw-degrading enzymes that could degrade different types of straws. For example, Swain et al. [35] found seven different *Trichoderma* spp. that could produce higher straw-degrading enzymes, such as cellulase, endoglucanase, xylanase, and laccase, thereby accelerating rice straw decomposition. Moreover, raw wheat straw was found to be efficiently hydrolyzed by *T. guizhouense* cellulase cocktail within 35 h. In the present study, the abundance of *Trichoderma* in both SM and SC treatments was significantly higher than that in the SR and SO treatments. Compared with SR and SO treatments, soil microorganisms had more chances to directly utilize chopped straws in SM and SC treatments, which indicated that *Trichoderma* in SM and SC treatments were more efficient in straw degradation compared with other two treatments, thereby increased the abundance of *Trichoderma*.

The present study also showed that *Chaetomium* was an important fungus that had significantly greater abundance in the soils with straw-returning than those with straw removal. The abundance of *Chaetomium* in SM treatments was significantly higher than in other three treatments, which indicated that *Chaetomium* could effectively degrade chopped straws on the surface of soil, and result in the high abundance in SM treatments. Several studies reported that *Chaetomium* was involved in straw degradation. Wang et al. [36] found that *C. globosum* could increase the degradation rate of rice straw. Moreover, a cellobiohydrolase gene *ctcel7* and an endo-1,4-β-xylanase gene *xylcg* were identified and cloned from *C. thermophilum* and *C. globosum*, respectively, and their expression proteins exhibited high hydrolysis and conversion effect on pretreated wheat straw [37,38]. Therefore, based on the role of *Chaetomium* in straw degradation and a higher abundance of *Chaetomium* in SM treatments than other treatments, we deduced that *Chaetomium* may be used as a potentially effective fungal agent for involving straw degradation in this study. Similar to *Trichoderma*, besides the function of straw-degrading, *Chaetomium* has been used in agriculture for its excellent effect on plant protection and growth promotion [39,40].

Besides fungus, some bacteria genera were also probably involved in straw degradation. *Streptomyces* had wildly application to agricultural crops for promoting plant growth, improving environmental resistance, as well as plant disease protection [41,42,43]. Studies reported that *Streptomyces* might participate in straw degradation. Xu et al. [44] found that *S. griseorubens* C-5 could enhance the enzymic activity of cellulase, laccase, peroxidase, xylanase and pectinase, and predicted that *S. griseorubens* could effectively decompose rice straw and improve the resource utilization of the rice straw. Another *S. griseorubens* strain JSD-1 also exhibited potential to degrade various chemical compositions of inoculated rice straw. More recent studies found that the cellulolytic enzymes produced by JSD-1 favored the conversion of rice straw [45,46]. In the present study, the abundance of *Streptomyces* in the soils with three straw-returning treatments was all significantly higher than in the soils with the straw removal treatment. Based on the role of *Streptomyces* in straw degradation, we deduced that *Streptomyces* might had the ability to utilize chopped straws in this study and thereby increased their abundance in all three straw-returning treatments, and could be further used as potential straw-degrading bacterial. Further elaboration on the specific roles of microorganisms within the community changes in this study would be beneficial.

Besides the potential ability of straw-degrading, some microorganisms, such as *Trichoderma*, *Chaetomium* and *Streptomyces*, play important roles in plant protection with benefits on controlling the incidence of various plant diseases [37,47,48]. In addition, several microorganisms were preliminarily screened had the potential to increase crop productivity. *Trichoderma* and *Bacillus* were two main microbial groups that had been reported to improve crop productivity. *Trichoderma* had been reported could increase the productivity of soybean, maize, wheat [49,50,51]. *Bacillus* strains also exhibited excellent ability to increase the productivity of crops such as rice, wheat, potato and maize [52,53,54,55]. Moreover, *Streptomyces* and *Metarhizium* strains had also been reported to be involved in crop productivity increasement. *Streptomyces* could increase soybean and rice productivity [56,57]. *Metarhizium* could increase wheat and rice productivity [58,59]. 

Isolation and screening of straw degradation related microbial agents from soil, and finally application on the field to degrade straw is very important for resource utilization and environmental protection. The commonly method used to apply straw degradation related microbial agents to the field is to prepare the microbial suspensions, and sprayed into the field to degrade straws. Zhao et al. sprayed 1.70 × 10^8^ CFU mL^−1^ bacterial solution together with straw in the field [60]. Therefore, in the future work, we could firstly screen straw degradation related microorganisms, such as *Trichoderma*, *Chaetomium*, and *Streptomyces*, and then sprayed the suspensions of these microorganisms to the field to degrade straws.

The present study also suggests the need of future work in several aspects. (1) Using the culture approach to isolate and cultivate microorganisms that were identified through Illumina MiSeq sequencing with high potential for straw degradation. (2) In vitro investigation on the straw degradation ability of the isolated microorganisms. (3) Enhance the straw degradation ability of objective microorganisms by optimizing the microbial cultivate conditions and culture medium composition through response surface method or orthogonal experiment. (4) Deep analyses of molecular mechanisms for straw degradation by selected microorganisms, and screening of relevant genes and investigation of their functions in straw degradation through gene knockout, complementary and overexpression. (5) Construction of gene engineering microbial strains that could improve the straw degradation ability. (6) Preparation and development of straw degradation microorganism products and application for straw degradation in the field. (7) Established the association between straw degradation related key microbial taxa and soil physical and chemical characters, and soil functions, such as soil enzyme activity, straw decomposition or nutrient cycling rate. (8) Investigate the dynamic changes of microbial community for each straw-returning treatment.

## 5. Conclusions

This study assessed the effects of three straw-returning methods (i.e., straw mulching, straw mulching and overturning, and straw crushed and mixed) on soil bacterial and fungal diversity and community composition as compared to straw removal as control. It was found that all straw-returning methods altered the soil microbial community composition and abundance as compared to straw removal. Notably, straw mulching treatment, and straw crushed and mixed treatment increased the diversity of both soil bacteria and fungi compared with straw mulching and overturning treatment. *Trichoderma*, *Chaetomium* and *Streptomyces* were preliminarily screened as microorganisms with high straw-degrading potential. More future work is needed for isolation and cultivation of the microorganisms, in vitro investigation of their straw degradation ability, optimization of the environmental conditions their application, further development of their potential for field applications, and established the association between straw degradation related key microbial taxa and soil functions. 

## Data Availability

The original contributions presented in this study are included in the article/Supplementary Material. Further inquiries can be directed to the corresponding authors.

## Supplementary Materials

The following supporting information can be downloaded at: https://www.mdpi.com/article/10.3390/microorganisms13081749/s1, Figure S1. Soil bacterial community composition at the phylum level. (A) SR, (B) SM, (C) SO, (D) SC. SR: straw removal; SM: straw mulching; SO: straw mulching and overturning; and SC: straw crushed and mixed. Figure S2. Soil fungal community composition at the phylum level. (A) SR, (B) SM, (C) SO, (D) SC. SR: straw removal; SM: straw mulching; SO: straw mulching and overturning; and SC: straw crushed and mixed.

## References

[1] Gao J., Zhu J., Huang Y.G., Zhang M., Zhang R.C., Peng Y.Q. (2019). Discussion on the utilization methods of straw. Crop Res..

[2] Li H.M., Zhang M., Li X., Nie M., Tian X.W. (2021). Research progress of straw degradation by microorganisms. Shandong Chem. Ind..

[3] Sun L.N., Ma X.Y., Liu K.B., Zheng X.H., Zhang H.L., Rong L.G. (2018). A Review on research advances on microbial treatment and strengthening techniques of crop straw. J. Shenyang Univ. (Nat. Sci.).

[4] Song P. (2018). Research progress of crop straw development and utilization. Mod. Anim. Husbandry.

[5] Huang L.X., Wang Y.J., Huang Q., Wei L., Li X., Chen W.S., Huang Y.F., Liu Z.Z. (2021). Effects of two ways of cornstalk returning on soil properties, growth and quality of sweet corn. Guangdong Agr. Sci..

[6] Gou Z., Yin W., Chai Q. (2021). Straw and residual film management enhances crop yield and weakens CO_2_ emissions in wheat-maize intercropping system. Sci. Rep..

[7] Chen L., Sun S., Yao B., Peng Y., Gao C., Qin T., Zhou Y., Sun C., Quan W. (2022). Effects of straw return and straw biochar on soil properties and crop growth: A review. Front. Plant Sci..

[8] Liu G.Y., He A.L., Du J., Yang Z.P., Pan X.Y., Xu J.D., Zheng N., Zhang Y.T. (2022). Effects of maize straw returning amount on soil enzyme activity, microbial biomass, and bacterial community in lime concretion black soil. J. Agr. Resour. Environ..

[9] Xu T. (2022). Effects of different ways of returning corn stalks to the field on corn growth characteristics, yield and its composition under maize bean rotation. Anhui Agri. Sci. Bull..

[10] Li J., Ren L.J., Li X.Y., Bi R.X., Jin X.X., Yu N., Zhang Y.L., Zhou H.T., Zhang Y.L. (2023). Effects of different straw returning patterns on soil CO_2_ emission and carbon balance in maize field. Sci. Agric. Sin..

[11] Wanmolee W., Sornlake W., Rattanaphan N., Suwannarangsee S., Laosiripojana N., Champreda V. (2016). Biochemical characterization and synergism of cellulolytic enzyme system from *Chaetomium globosum* on rice straw saccharification. BMC Biotechnol..

[12] Saritha M., Arora A., Singh S., Nain L. (2013). *Streptomyces griseorubens* mediated delignification of paddy straw for improved enzymatic saccharification yields. Bioresour. Technol..

[13] Chen K.J., Tang J.C., Xu B.H., Lan S.L., Cao Y. (2019). Degradation enhancement of rice straw by co-culture of *Phanerochaete chrysosporium* and *Trichoderma viride*. Sci. Rep..

[14] Mei J., Shen X., Gang L., Xu H., Wu F., Sheng L. (2020). A novel lignin degradation bacteria-*Bacillus amyloliquefaciens* SL-7 used to degrade straw lignin efficiently. Bioresour. Technol..

[15] Wang L., Guan H., Hu J., Feng Y., Li X., Yusef K.K., Gao H., Tian D. (2022). *Aspergillus niger* enhances organic and inorganic phosphorus release from wheat straw by secretion of degrading enzymes and oxalic acid. J. Agric. Food Chem..

[16] Liu Z.G., Lu H.B., Zhao H.C., Wang J.Q., He J.P., Wang Y.Q., Huang Z.H., Zhao H.X., Wei D. (2022). Effects of methods for spring maize straw-returning to field on soil microbial biomas C, N, P and enzyme activities in dry farming area. Acta Agr. Boreali-Occident. Sin..

[17] Yang H., Zhao Y., Ma J., Rong Z., Chen J., Wang Y., Zheng X., Ye W. (2022). Wheat straw return influences soybean root-associated bacterial and fungal microbiota in a wheat-soybean rotation system. Microorganisms.

[18] Xu N., Tan G., Wang H., Gai X. (2016). Effect of biochar additions to soil on nitrogen leaching, microbial biomass and bacterial community structure. Eur. J. Soil Biol..

[19] Wen Y.C., Li H.Y., Lin Z.A., Zhao B.Q., Sun Z.B., Yuan L., Xu J.K., Li Y.Q. (2020). Long-term fertilization alters soil properties and fungal community composition in fluvo-aquic soil of the North China Plain. Sci. Rep..

[20] Chen S., Zhou Y., Chen Y., Gu J. (2018). fastp: An ultra-fast all-in-one FASTQ preprocessor. Bioinformatics.

[21] Li F., Hitch T.C.A., Chen Y., Creevey C.J., Guan L.L. (2019). Comparative metagenomic and metatranscriptomic analyses reveal the breed effect on the rumen microbiome and its associations with feed efficiency in beef cattle. Microbiome.

[22] Magoč T., Salzberg S.L. (2011). FLASH: Fast length adjustment of short reads to improve genome assemblies. Bioinformatics.

[23] Edgar R.C. (2013). UPARSE: Highly accurate OTU sequences from microbial amplicon reads. Nat. Methods.

[24] Lan Y., Wang Q., Cole J.R., Rosen G.L. (2012). Using the RDP classifier to predict taxonomic novelty and reduce the search space for finding novel organisms. PLoS ONE.

[25] Segata N., Izard J., Waldron L., Gevers D., Miropolsky L., Garrett W.S., Huttenhower C. (2011). Metagenomic biomarker discovery and explanation. Genome Biol..

[26] Miguel M.A., Kim S.H., Lee S.S., Cho Y.I. (2021). Impact of soil microbes and oxygen availability on bacterial community structure of decomposing poultry carcasses. Animals.

[27] Murase J., Takenouchi Y., Iwasaki K., Kimura M. (2014). Microeukaryotic community and oxygen response in rice field soil revealed using a combined rRNA-gene and rRNA-based approach. Microbes Environ..

[28] Castaño C., Lindahl B.D., Alday J.G., Hagenbo A., Martínez de Aragón J., Parladé J., Pera J., Bonet J.A. (2018). Soil microclimate changes affect soil fungal communities in a Mediterranean pine forest. New Phytol..

[29] Bandopadhyay S., Martin-Closas L., Pelacho A.M., DeBruyn J.M. (2018). Biodegradable plastic mulch films: Impacts on soil microbial communities and eosystem functions. Front. Microbiol..

[30] Bradford M.A., Tordoff G.M., Eggers T., Jones T.H., Newington J.E. (2002). Microbiota, fauna, and mesh size interactions in litter decomposition. Oikos.

[31] Zhang F.T., Wang T.S., Jiang H., Zhang B., Han Y., Yao S.H. (2024). Effects of size and amount and burial depth on early decomposition of maize straw and the links to microbial and nematode communities in the Mollisol of China. Soil Use Manag..

[32] Abdenaceur R., Farida B.T., Mourad D., Rima H., Zahia O., Fatma S.H. (2022). Effective biofertilizer *Trichoderma* spp. isolates with enzymatic activity and metabolites enhancing plant growth. Int. Microbiol..

[33] Ren X., Branà M.T., Haidukowski M., Gallo A., Zhang Q., Logrieco A.F., Li P., Zhao S., Altomare C. (2022). Potential of *Trichoderma* spp. for biocontrol of aflatoxin-producing *Aspergillus flavus*. Toxins.

[34] TariqJaveed M., Farooq T., Al-Hazmi A.S., Hussain M.D., Rehman A.U. (2021). Role of *Trichoderma* as a biocontrol agent (BCA) of phytoparasitic nematodes and plant growth inducer. J. Invertebr. Pathol..

[35] Swain H., Adak T., Mukherjee A.K., Sarangi S., Samal P., Khandual A., Jena R., Bhattacharyya P., Naik S.K., Mehetre S.T. (2021). Seed biopriming with *Trichoderma* strains isolated from tree bark improves plant growth, antioxidative defense system in rice and enhance straw degradationcapacity. Front. Microbiol..

[36] Wang Z., Cui J., Gao W., Yang Q., Chen L., Yang L., Sun Q., Zhang H. (2020). Effects of rice straw structure on chaetoglobosin A production by *Chaetomium globosum* CGMCC 6882. Int. J. Biol. Macromol..

[37] Han C., Yang R.R., Sun Y.X., Liu M.Y., Zhou L.F., Li D.C. (2020). Identification and characterization of a novel hyperthermostable bifunctional cellobiohydrolase- xylanase enzyme for synergistic effect with commercial cellulase on pretreated wheat straw degradation. Front. Bioeng. Biotechnol..

[38] Singh R.K., Tiwari M.K., Kim D., Kang Y.C., Ramachandran P., Lee J.K. (2013). Molecular cloning and characterization of a GH11 endoxylanase from *Chaetomium globosum*, and its use in enzymatic pretreatment of biomass. Appl. Microbiol. Biotechnol..

[39] Tian Y., Fu X., Zhang G., Zhang R., Kang Z., Gao K., Mendgen K. (2022). Mechanisms in growth-promoting of cucumber by the endophytic fungus *Chaetomium globosum* strain ND35. J. Fungi.

[40] Zhao S.S., Zhang Y.Y., Yan W., Cao L.L., Xiao Y., Ye Y.H. (2017). *Chaetomium globosum* CDW7, a potential biological control strain and its antifungal metabolites. FEMS Microbiol. Lett..

[41] Gao W.L., Fang J.L., Zhu C.Y., Xu W.F., Lyu Z.Y., Chan X.A., Zhao Q.W., Li Y.Q. (2023). Identification and characterization of a new regulator, TagR, for environmental stress resistance based on the DNA methylome of *Streptomyces* roseosporus. Microbiol. Spectr..

[42] Le K.D., Yu N.H., Park A.R., Park D.J., Kim C.J., Kim J.C. (2022). *Streptomyces* sp. AN090126 as a biocontrol agent against bacterial and fungal plant diseases. Microorganisms.

[43] Vurukonda S.S.K.P., Giovanardi D., Stefani E. (2018). Plant growth promoting and biocontrol activity of *Streptomyces* spp. as endophytes. Int. J. Mol. Sci..

[44] Xu J., Yang Q. (2010). Isolation and characterization of rice straw degrading *Streptomyces griseorubens* C-5. Biodegradation.

[45] Feng H., Sun Y., Zhi Y., Mao L., Luo Y., Wei X., Zhou P. (2015). Lignocellulose degradation by the isolate of *Streptomyces griseorubens* JSD-1. Funct. Integr. Genom..

[46] Zhang D., Luo Y., Chu S., Zhi Y., Wang B., Zhou P. (2016). Biological pretreatment of rice straw with *Streptomyces griseorubens* JSD-1 and its optimized production of cellulase and xylanase for improved enzymatic saccharification efficiency. Prep. Biochem. Biotechnol..

[47] Lahlali R., Ezrari S., Radouane N., Kenfaoui J., Esmaeel Q., El Hamss H., Belabess Z., Barka E.A. (2022). Biological control of plant pathogens: A global perspective. Microorganisms.

[48] Khan S., Srivastava S., Karnwal A., Malik T. (2023). *Streptomyces* as a promising biological control agents for plant pathogens. Front. Microbiol..

[49] Marra R., Lombardi N., d’Errico G., Troisi J., Scala G., Vinale F., Woo S.L., Bonanomi G., Lorito M. (2019). Application of Trichoderma strains and metabolites enhances soybean productivity and nutrient content. J. Agric. Food Chem..

[50] Mitrović I., Čanak P., Tančić Živanov S., Farkaš H., Vasiljević M., Ćujić S., Zorić M., Mitrović B. (2025). *Trichoderma harzianum* in biocontrol of maize fungal diseases and relevant mycotoxins: From the laboratory to the field. J. Fungi.

[51] Ali I., Khan A., Ali A., Ullah Z., Dai D.Q., Khan N., Khan A., Al-Tawaha A.R., Sher H. (2022). Iron and zinc micronutrients and soil inoculation of *Trichoderma harzianum* enhance wheat grain quality and yield. Front. Plant Sci..

[52] Pandey N., Vaishnav R., Rajavat A.S., Singh A.N., Kumar S., Tripathi R.M., Kumar M., Shrivastava N. (2024). Exploring the potential of *Bacillus* for crop productivity and sustainable solution for combating rice false smut disease. Front. Microbiol..

[53] Zahra S.T., Tariq M., Abdullah M., Azeem F., Ashraf M.A. (2023). Dominance of *Bacillus* species in the wheat (*Triticum aestivum* L.) rhizosphere and their plant growth promoting potential under salt stress conditions. PeerJ.

[54] Tahir M., Ahmad I., Shahid M., Shah G.M., Farooq A.B.U., Akram M., Tabassum S.A., Naeem M.A., Khalid U., Ahmad S. (2019). Regulation of antioxidant production, ion uptake and productivity in potato (*Solanum tuberosum* L.) plant inoculated with growth promoting salt tolerant *Bacillus* strains. Ecotoxicol. Environ. Saf..

[55] de Oliveira-Paiva C.A., Bini D., de Sousa S.M., Ribeiro V.P., Dos Santos F.C., de Paula Lana U.G., de Souza F.F., Gomes E.A., Marriel I.E. (2024). Inoculation with *Bacillus megaterium* CNPMS B119 and *Bacillus subtilis* CNPMS B2084 improve P-acquisition and maize yield in Brazil. Front. Microbiol..

[56] Villafañe D.L., Maldonado R.A., Bianchi J.S., Kurth D., Gramajo H., Chiesa M.A., Rodríguez E. (2024). *Streptomyces* N2A, an endophytic actinobacteria that promotes soybean growth and increases yield and seed quality under field conditions. Plant Sci..

[57] Gao Y., Ning Q., Yang Y., Liu Y., Niu S., Hu X., Pan H., Bu Z., Chen N., Guo J. (2021). Endophytic *Streptomyces hygroscopicus* OsiSh-2-mediated balancing between growth and disease resistance in host rice. mBio.

[58] Bhuiyan A.U., Chowdhury M.Z.H., Mim M.F., Siddique S.S., Haque M.A., Rahman M.S., Islam S.M.N. (2024). Seed priming with *Metarhizium anisopliae* (MetA1) improves physiology, growth and yield of wheat. Heliyon.

[59] Chowdhury M.Z.H., Mostofa M.G., Mim M.F., Haque M.A., Karim M.A., Sultana R., Rohman M.M., Bhuiyan A.U., Rupok M.R.B., Islam S.M.N. (2024). The fungal endophyte *Metarhizium anisopliae* (MetA1) coordinates salt tolerance mechanisms of rice to enhance growth and yield. Plant Physiol. Biochem..

[60] Zhao S., Wang H., Wang J. (2023). Synthesis and application of a compound microbial inoculant for effective soil remediation. Environ. Sci. Pollut. Res Int..

