# Supplementary material for: Varying Effects of Straw-Returning Methods on Soil Microbial Diversity and Community Composition in Northeast China
Source: Microorganisms. 2025 Jul 26;13(8):1749. doi: 10.3390/microorganisms13081749 (PMC12388232; doi:10.3390/microorganisms13081749)
Supplement: Supplementary file 1 [file microorganisms-13-01749-s001.zip › microorganisms-3714723-supplementary.pdf]

Varying Effects of Straw-Returning Methods on Soil Microbial Diversity and Community Composition in Northeast China

Yitao Zhang [1], Yuxian Wang [2]* and Zhanbin Sun [3]*

[1] Institute of Geographic Sciences and Natural Resources Research, Chinese Academy of Sciences,
Beijing 100101, China; zhangyt@igsnrr.ac.cn
[2] Qiqihar Branch of Heilongjiang Academy of Agricultural Sciences, Qiqihar 161006, China
[3] School of Light Industry Science and Engineering, Beijing Technology and Business University,
Beijing 100048, China
* Correspondence: wyx13836209470@163.com (Y.W.); twins5616@126.com (Z.S.)

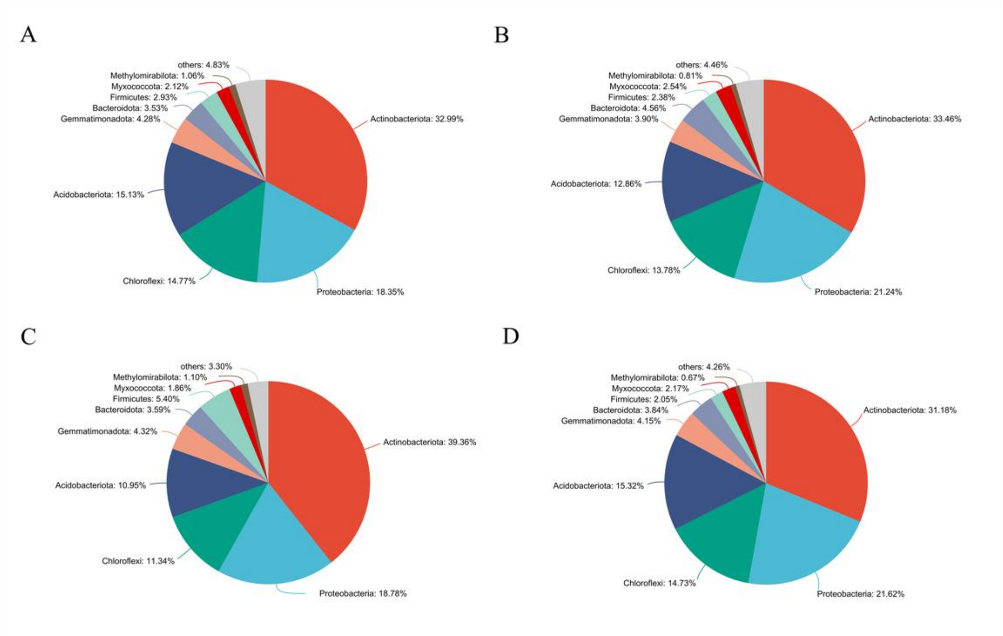

Figure S1. Soil bacterial community composition at the phylum level. (A) SR, (B) SM, (C) SO, (D) SC. SR: straw removal; SM: straw mulching; SO: straw mulching and overturning; and SC: straw crushed and mixed.

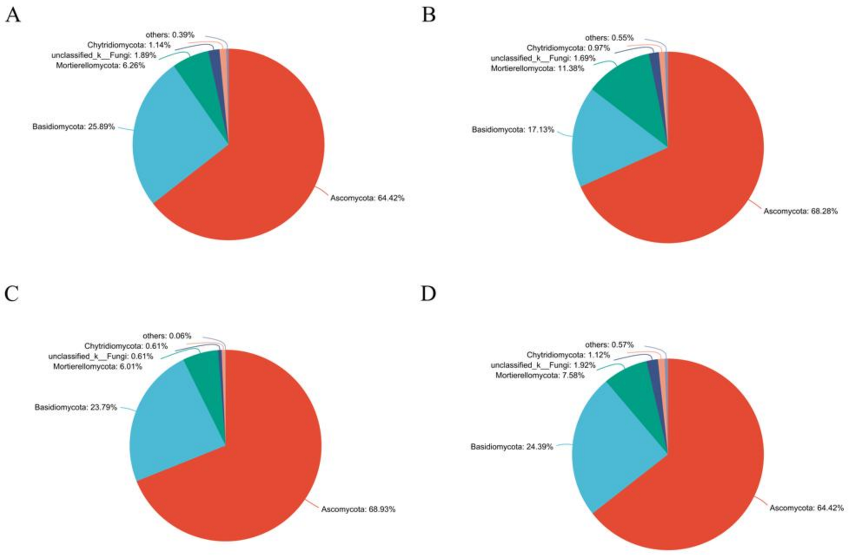

Figure S2. Soil fungal community composition at the phylum level. (A) SR, (B) SM, (C) SO, (D) SC. SR: straw removal; SM: straw mulching; SO: straw mulching and overturning; and SC: straw crushed and mixed.
